# When patients and surgeons disagree about surgical outcome: investigating patient factors and chart note communication

**DOI:** 10.1186/s12955-015-0343-0

**Published:** 2015-09-29

**Authors:** Carolyn E. Schwartz, Armon Ayandeh, Joel A. Finkelstein

**Affiliations:** DeltaQuest Foundation, Inc., 31 Mitchell Road, Concord, MA 01742 USA; Departments of Medicine and Orthopaedic Surgery, Tufts University Medical School, Boston, MA USA; Division of Orthopaedics, Sunnybrook Health Sciences Center and the University of Toronto, 2075 Bayview Avenue, Room MG361, Toronto, ON Canada

**Keywords:** Communication, Patient-reported outcomes, Clinical outcomes, Mismatch, Response shift, Medical record, Disability

## Abstract

**Objective:**

Effective physician-patient communication is a critical component of a clinical practice and in order to achieve optimal patient outcomes. We aimed to investigate indirect effects of physician-patient communication by examining the relationship between a physician-patient mismatch in perceived outcomes and content in the medical record’s clinical note. We compared patient records whose perceived subjective assessment of surgery outcomes agreed or disagreed with the surgeon's perception of that outcome (Subjective Disagreement).

**Methods:**

This study included 172 spine surgery patients at a teaching hospital. Patient-reported outcomes included the Oswestry Disability Index; the Short-Form 36; and a Visual Analogue Scale items for leg and back pain. We content-analyzed the clinical note in the medical record, and used logistic regression to evaluate predictors of Subjective Disagreement (*n* = 41 disagreed vs. 131 agreed).

**Results:**

Patient and surgeon agreed in 76 % of cases and disagreed in 24 % of cases. Patients who assessed their outcome worse than their surgeons tended to be less educated and involved in litigation. They also tended to report worsened mental health and leg pain. Content analysis revealed group differences in surgeon communication patterns in the chart notes related to how symptom change was emphasized, how follow-up was described, and a specific word reference. Specifically, disagreement was predicted by using “much” to emphasize the findings and noting long-term prognosis. Agreement was predicted by use of positive emphasis terms, having an “as-needed” follow-up plan, and using “happy” in the chart note.

**Conclusion:**

The nature of measuring outcomes of surgery is based on patient perception. In surgeon-patient perspective mismatches, patient factors may serve as barriers to improvement. Worsened change on patient-reported mental health may be an independent factor which colors the patient’s general perceptions. This aspect of treatment may be missed by the spine surgeon. Chart note communication styles reflect the subjective disagreement. Investigating and/ or treating mental health deterioration may be valuable in resolving this mismatch and for overall outcome.

**Electronic supplementary material:**

The online version of this article (doi:10.1186/s12955-015-0343-0) contains supplementary material, which is available to authorized users.

## Introduction

The art and science of medicine has needed to evolve in response to improvements in technology, patient expectations, and an increasingly litigious world. The training of future physicians has likewise evolved, and curricula sanctioned by governing bodies have helped maintain a high standard of care. One such body, the Canadian Medical Education Directives for Specialists (CanMEDS) [1, 2], is a competency-based framework that has been adopted to guide training programs and evaluations of physicians and specialists. The communicator role is a particularly essential aspect of this framework for establishing rapport and trust, formulating a diagnosis, delivering information, striving for mutual understanding, and facilitating a shared plan of care. Effective communication is critical for optimal patient outcomes.

**Fig. 1:**
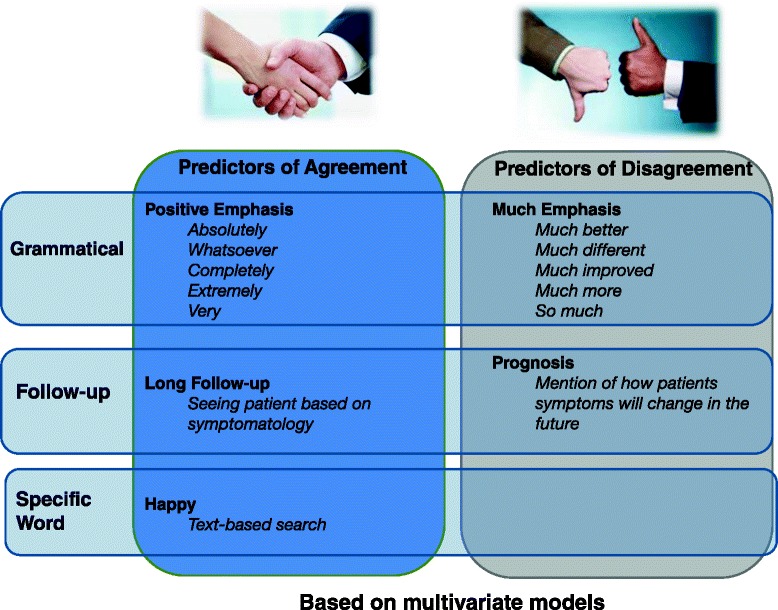
Content analysis revealed group differences in surgeon communication patterns in the chart notes related to how symptom change was emphasized, how follow-up was described, and a specific word reference. Specifically, disagreement was predicted by using “much” to emphasize the findings and noting long-term prognosis. Agreement was predicted by use of positive emphasis terms, having an “as-needed” follow-up plan, and using “happy” in the chart note

Central to effective communication is being able to convey orally to the patient realistic expectations about surgical outcome, as well as effective written information about a medical encounter [3]. The purpose of this study was to examine predictors of physician-patient mismatch in perceived outcomes of spine surgery. We compared patient records whose perceived subjective assessment of surgery outcomes agreed or disagreed with the surgeon's perception of that outcome, and investigated demographic factors, patient-reported outcomes, and the clinical chart note content in the medical record.

## Materials and methods

### Sample and design

This prospective study included patients who had undergone one or two level decompression surgery; (discectomy or laminectomy) with leg-dominant pain. Multilevel decompressions and fusion procedures that require longer rehabilitation until maximum recovery were excluded. Excluding patients with more complicated operations was done to create a homogenous group of patients Study participants were consecutively recruited from the practices of three spine surgeons at a major teaching hospital in Canada. As standard protocol, surgeons engaged all patients in a transparent discussion about what to expect from the surgery. The surgeons explained what the surgery will improve and will not improve, i.e. leg pain will be better, back pain may or may not. Patients provided baseline data pre-surgically, and follow-up data post-surgery. The study protocol was approved by the Sunnybrook Health Sciences Centre Research Ethics Board, and all participants provided written informed consent. Participants completed self-report questionnaires pre-surgery and at first follow-up.

### Measures

Three categories of measures were used in this study. *Demographic characteristics* were collected from the patients, including age, gender, duration of symptoms, employment status (working at present, retired, student, homemaker), smoking status (i.e., current smoker or not), and associated co-morbid health conditions and other musculoskeletal conditions. We also tracked *having an incentive not to work*. This variable was characterized as involvement in compensation or litigation that would serve as an incentive not to experience symptom improvement over time (e.g., currently on disability or worker’s compensation, or involved in litigation related to their illness or injury.

*Standardized spine outcome measures* were collected in this study: (1) the generic Short-Form-36 v1 (SF-36v1) [4] comprising eight domains assessing evaluative functional health, with higher scores reflecting better functional health; (2) two Likert-scaled visual analogue scale (VAS) items measuring back and leg pain on a 100-point scale, with higher scores indicating worse pain [2]; (3) The 10-item disease specific Oswestry Disability Index (ODI) [1] measuring perceived pain during activities of daily living.

*Clinical chart notes* were also utilized in this study. These notes included data from the neurological examination (e.g., straight leg raising, numbness, strength, walking distance); as well as the recorded subjective assessment of surgical outcome from patient and surgeon. Patients were asked how they would characterize their surgical outcome (poor, moderate, excellent). Surgeons were asked to note in the chart how they would characterize the patient’s outcome based on the objective examination, as well as their understanding of the patient’s change in function. Surgeons categorized this understanding as not improved, not fully improved, or fully improved. The chart note also provided information on whether there were complications from the surgery; and reported symptoms of leg or back pain. Additionally, written summaries of the patient’s follow-up appointment were captured verbatim. These included documentation for the patient’s medical record as well as communications to other health care providers.

### Content analysis

Text from the clinical chart notes were content analyzed using QSR NVIVO 10 [5]. Two independent raters read and coded all chart notes for terms or concepts that were identified after coding an initial 100 patients (see Additional file 1 for complete listing and explanation of nodes). After all records were coded by both raters, inter-rater reliability was computed using the kappa coefficient [6]. It was greater than 90 % on most nodes. Adjudication then took place such that all differences in codes were discussed to determine the most appropriate coding for the record. This process resulted in 100 % inter-rater reliability.

### Statistical analysis

Patients were characterized on the basis of whether their subjective assessment of surgery outcome was similar or disagreed with the surgeon’s assessment. We then used this grouping variable (Subjective Agreement vs Subjective Disagreement) as the dependent variable in a series of hierarchical logistic regression analyses. We began with univariate regressions within a class of variables (i.e., demographic, patient-reported outcome, clinical-chart nodes). We then computed multivariate models within a class of variables. We did not combine the three classes of variables into a single multivariate model, due to sample size constraints and the resulting limited power. We thus present the results of the three sets of models in terms of triangulating on the prediction of the grouping variable. The Type I error rate for the univariate models was *p* < 0.10, and *p* < 0.05 for the multivariable model, as per standard hierarchical modeling approaches. Stata 13 [7] was used for logistic regression modeling.

## Results

Table 1 shows how the sample was characterized in terms of subjective assessment of surgical outcome. There were 130 patients (76 %) whose subjective assessment agreed with the surgeon’s, one who thought the outcome was better than the surgeon did, and 41 (24 %) who thought they did worse than the surgeon thought. For the purpose of the subsequent analyses, we dropped the one outlier who thought s/he did better than the surgeon thought because this person would have been qualitatively different than both those who agreed and those who disagreed with their surgeon’s assessment.

**Table 1:** Characterization of Patient-Surgeon Agreement on Surgery Outcome^a^

	Patient's perspective			
Surgeon's perspective	*Poor*	*Moderate*	*Excellent*	*Total*
*Not improved*	3	**1**	0	4
*Not fully improved*	**9**	2	0	11
*Fully improved*	0	**32**	125	157
*Total*	12	35	125	172

^*a*^ *Bolded values represent discrepancy groups*

The remaining 171 patients had a mean post-operative follow-up of 7.7 weeks (SD = 3.2). The procedures which were the basis of the study would be expected to result in significant recovery by this time point as the operative morbidity rates are generally low. Table 2 provides the descriptive statistics on the demographics, comorbidities, and baseline patient-reported outcome scores for the 171 people who were retained in the analysis.

**Table 2:** Sample Demographics

Variable	*N* = 171 ( % or SD)
Time points	
Mean Weeks of Follow-up (SD)	7.73 (3.23)
Gender: *N* (%)	
Male	80 (46.78)
Female	60 (35.09)
Missing	31 (18.13)
Surgical Diagnosis^a^: *N* (%)	
Disc Herniation	97 (56.73)
Spinal Stenosis	69 (40.35)
Spondylolithesis	29 (16.96)
Other	8 (4.68)
Co-morbidities: *N* (%)	
Depression	14 (8.19)
Cardiac Conditions	11 (6.43)
Diabetes	13 (7.60)
Thyroid Conditions	5 (2.92)
Cancer	5 (2.92)
Pulmonary Conditions	4 (2.08)
Stroke	2 (1.17)
Peripheral Neuropathy	2 (1.17)
Other	19 (11.11)
Education: *N* (%)	
Less than High School	12 (7.02)
Graduated From High School or GED	23 (13.45)
Some College or Technical School	28 (16.37)
Graduated from College	30 (17.54)
Postgraduate School or Degree	39 (22.81)
Missing	39 (22.81)
Employment Status at Pre-surgical Baseline^b^: *N* (%)	
Working	56 (32.75)
On leave of absence	11 (6.43)
Unemployed	6 (3.51)
Retired	42 (24.56)
Disabled	17 (9.94)
Homemaker	6 (3.51)
Student	5 (2.92)
Other	15 (8.77)
Missing	17 (9.94)
Pain Medication Use at Pre-surgical Baseline: *N* (%)	
Use Narcotics	37 (21.64)
Current Smoker	39 (22.81)
Age: Mean Years (SD)	51.95 (16.59)
Range	[20–84]
Pre-surgical Baseline Patient-Reported Outcome Scores: Mean (SD)	
SF-36 PCS	32.68 (8.19)
SF-36 MCS	43.91 (12.84)
VAS Back	54.78 (30.43)
VAS Leg	65.36 (26.45)
ODI	24.58 (10.96)

^a^6 people had more than one diagnosis; 92 people had no diagnosis

^b^3 people reported more than 1 category

### Demographic predictors of subjective disagreement

Multivariate logistic models suggested that having a subjective assessment of outcome that was worse than their surgeon’s was more likely among people who had lower education (OR = 0.20, *p* < 0.01), and who had an incentive not to work (OR = 4.3, *p* < 0.01) (Table 3). There was no impact of age, gender, comorbidities, or pre-surgical use of narcotic analgesic, or pre-surgical smoking (Appendix).

**Table 3:** Logistic regression model of significant demographic factors predicting disagreement between doctor and patient

	Odds Ratio	Std. Err.	z	*P*-value	95 % Conf.	Interval	*n*	Pseudo r2
**Demographic Factors Model**				0.0058 ^*^			83	0.14
Education Bin	0.20	0.13	−2.57	0.01	0.060	0.685		
NoWorklncentive	4.31	2.40	2.61	0.009	1.44	12.87		
Smoking								
Never Smoked	Referent							
Quit over a year ago	0.30	0.19	−1.85	0.064	0.083	1.073		
Current Smoker	0.24	0.18	−1.87	0.061	0.053	1.071		

^*^ *P*-value specified for the model is based on a chi2 test, whereas individual item *p*-values are calculated from the z-statistic

### Patient-reported outcome predictors of subjective disagreement

Multivariate models suggested that patients whose subjective assessment of outcome was worse than their surgeons tended to report worsened mental health component scores on the SF-36, and worsened VAS leg pain (OR = 0.94 and 1.03, *p* < 0.05 and 0.02, respectively) (Table 4). There was no impact of change in VAS back pain, physical component score of the SF-36, or ODI (Table 4).

**Table 4:** Logistic regression model of significant patient-reported outcomes predicting disagreement between doctor and patient

	Odds Ratio	Std. Err.	z	*P*-value	95 % Conf.	Interval	*n*	Pseudo r2
**Patient-reported Outcomes Model**				0.0001^*^			84	0.24
VAS Back Change	1.00	0.01	0.42	0.68	0.98	1.03		
VAS Leg Change	1.03	0.01	2.38	0.017	1.00	1.05		
MCS Change	0.94	0.03	−2.00	0.046	0.88	1.00		
PCS Change	1.00	0.05	0.08	0.94	0.92	1.10		
ODI Change	1.02	0.04	0.41	0.68	0.94	1.10		

^*^ *P*-value specified for the model is based on a chi2 test, whereas individual item *p*-values are calculated from the z-statistic

### Clinical-chart node predictors of subjective disagreement

Multivariate logistic models of clinical chart-note coded nodes revealed that when there was disagreement between the perceived outcomes, surgeons were more likely to use “much”-emphasis terms (e.g., much better, much different, much improved, much more, so much) and to discuss prognosis of presenting symptoms (e.g. any mention of how patients symptoms will change in the future), that is that they would improve with time (OR = 5.5 and 4.5, respectively; *p* < 0.02 for both) (Table 5 and Fig. 1). Chart note predictors of patient-surgeon agreement included using positive language to emphasize the findings (e.g., absolutely, whatsoever, completely, extremely, very) (OR = 0.20, *p* < 0.02); having a follow-up plan that was open-ended (i.e., only if the patient presented with new or worsened symptoms) (OR = 0.06, *p* < 0.02), and the use of the term “happy” in the chart note (OR = 0.15, *p* < 0.04). There was no impact of factors such as re-engaging in activities of daily living, having an action plan, negative emphasis terms, symptoms, short follow-up plans, or other specific communication patterns coded (see Appendix for univariate analyses, and Additional file 1 for full listing of chart note codes).

**Table 5:** Logistic regression model of significant clinical chart notes predicting disagreement between doctor and patient

	Odds Ratio	Std. Err.	z	*P*-value	95 % Conf.	Interval	*n*	Pseudo r2
**Clinical Chart Notes Model**				<0.0001^*^			171	0.3813
Positive Emphasis terms	0.20	0.13	−2.46	0.014	0.053	0.719		
Much Emphasis terms	5.51	3.71	2.54	0.011	1.47	20.59		
All Emphasis terms	0.41	0.27	-1.34	0.18	0.11	1.51		
All Juxtaposition terms	1.17	0.57	0.31	0.75	0.45	3.05		
All Analgesics	2.19	1.57	1.09	0.28	0.54	8.92		
Discomfort	4.22	3.20	1.9	0.058	0.95	18.68		
Prognosis	4.46	2.65	2.51	0.012	1.39	14.32		
Follow-up only if symptoms	0.058	0.068	−2.45	0.014	0.0060	0.57		
Improve Stemmed	1.24	0.67	0.39	0.70	0.43	3.57		
Happy	0.15	0.13	−2.14	0.032	0.026	0.85		
Patient Discharged	3.53	3.85	1.16	0.25	0.42	29.96		

^*^ *P*-value specified for the model is based on a chi2 test, whereas individual item *p*-values are calculated from the z-statistic

## Discussion

Our results suggest that patient-surgeon perspective mismatches may relate to layers of factors, beginning with patient characteristics, continuing through patient-reported outcomes, and ending up as meta-messages (i.e., reading between the lines) transmitted in the medical record. The subtlety of these findings underscores the value of a qualitative analysis. Such patterns were detected only after careful coding and pattern analysis using a method that combined qualitative and quantitative techniques. This mixed-methods approach results in findings that would not be apparent from a simple reading of the text.  They are only revealed by dint of careful data analysis.

Lower levels of education, which can reflect low health literacy, were predictive of disagreement with their surgeon. Being involved in any litigation related to their spinal disorder (i.e., worker’s compensation, disability, or other litigation) also served as a risk factor for disagreement. Our findings underscore the potential bias in the self-report of patients in secondary-gain situations [6, 7], such as worker’s compensation and litigation. Our data suggest that when people are in a situation where they benefit from not getting better, their answers to patient-reported outcome questionnaires may not be valid. No measure, no matter how well it has demonstrated reliability and validity, can counteract the influence of secondary gain. Our findings are reminiscent of early work by Hayes and colleagues documenting that psychometric test results are unreliable among patients with nonorganic signs [8].

An unexpected finding was that the patient’s reporting worsened mental health or worsened leg pain, (as opposed to no change in leg pain) after surgery were significant factors in subjective disagreement. It should be noted that the basis of the surgeon’s assessment is on both objective and subjective grounds; including findings on the examination, notably the presence or absence of pain on straight leg-raising.

Our content analysis revealed subtle differences in how emphasis-language was used that differentiated patients whose subjective assessment differed from their surgeons. In addition to these differing ways of emphasizing their clinical findings, the chart text differed in how long-term follow-up was described. Whereas patients whose subjective assessment agreed with their surgeons were more likely to have non-specific follow-up planned (i.e., on an as-needed basis), those who disagreed with their surgeons were more likely to have chart notes that suggested that symptoms would improve with time without mention of a specific plan for a medical encounter. Finally, those whose subjective assessment agreed with their surgeons were more likely to have the term “happy” in their medical record.

Since the clinical chart note is a largely codified document, there are many terms and content domains that must be mentioned. It is thus not surprising that many relevant content areas did not differentiate the patient groups. For example, re-engagement in activities of daily living, having an action plan, and utilizing physical therapy are all expected aspects of surgical follow-up. These were not terms or content that differentiated our groups.

The clinical record may be saying more than the written word expresses, and it is possible the surgeon may be ill-prepared to appreciate the causes of the mismatch. The deterioration in mental health may be a harbinger of other personal or social factors in the patient’s life. The worsening of mental health would not be an expected outcome from the surgery itself, but may be related to external influences (e.g., increased interpersonal conflict due to financial or marital strain related to being unwell). It may also reflect poor adaptation to the patient’s new status quo. Mental health deterioration can lead to a negative coloring on all the subjective parameters of outcome, including leg pain, which was a predictor of subjective mismatch. Our findings suggest that measuring mental health status over time is not only important for understanding the patient’s well-being, but may also help to elucidate subjective-mismatch situations. On the basis of change in patient-reported measures of mental health, an appropriate referral can be made.

The implications of our findings for improving clinical outcomes of spine surgery might focus on interventions that focus on improving health literacy and insight. Such an intervention might focus on adjusting patient expectations of surgical outcomes to be more consistent with the likely outcome, and on increasing their insight into the negative impact of litigation/compensation on their health and well-being. The impact of such interventions on the eventual chart-note and surgeon-patient communication would be a useful path for future research.

The limitations of the present work should be noted. Whereas we chose a short follow-up time period that would be reflective of the pathology and treatment studied, a longer follow-up would be of more value except that the generic nature of patient-reported outcomes can introduce other biases due to changing life events that are unrelated to the surgery. There is also a possible bias introduced by missing data. Indeed, missing data issues restricted our ability to consider all classes of factors in one multivariate model because the sample size was substantially reduced when we did so. Further, our study included patients from a small number of surgeons (3) and from a country with notable socialized healthcare (Canada), both factors that may limit the generalizability of our findings.

Future work might continue our study’s line of research by replicating its delineation of patterns associated with surgeon-patient mismatch using PROs, content analysis of clinical chart notes, and demographic factors along with collecting a information on nonorganic signs (e.g., the Waddell Nonorganic Signs Test [9]).

## Conclusions

In summary, our findings underscore the multiple dimensions involved in surgeon-patient disagreement about subjective outcomes of spine surgery. In our study, this disagreement was apparent in about one quarter of the patients. Deterioration in the patient’s mental health score was a predictor of subjective disagreement, a context which may color the overall perception of the patient. The surgeon should be mindful of this, and may be in a position to facilitate other forms of support for their patients. This problem is worthy of further research to further characterize risk factors, and to investigate approaches to intervene at multiple levels to prevent disagreement and improve overall satisfaction with and outcomes of spine surgery.

## Appendix

**Table 6:** Univariate Logistic Regression Model Results

Variable	Odds Ratio	Std. Err.	z	*P* > |z|	95 % Conf. Interval		*n*	Pseudo r2
**Demographics**								
Age	1.00	0.01	−0.36	0.72	0.97	1.02	139	0.00
Gender	1.02	0.40	0.06	0.96	0.48	2.18	140	0.00
Education	0.76	0.11	−1.85	0.064	0.57	1.02	132	0.02
Comorbidities	1.24	0.43	0.62	0.54	0.63	2.46	85	0.00
Daily Occupation (e.g., employed, student, homemaker)	0.94	0.37	−0.17	0.87	0.43	2.02	141	0.00
Having No Work Incentive	3.38	1.64	2.51	0.012	1.31	8.73	96	0.05
Narcotic Usage	1.29	0.59	0.55	0.58	0.53	3.14	91	0.00
Smoking	1.59	0.41	1.78	0.075	0.96	2.63	137	0.02
**Patient-Reported**								
VAS Back Change	1.01	0.01	1.87	0.062	1.00	1.03	107	0.03
VAS Leg Change	1.04	0.01	4.34	0.0001	1.02	1.06	106	0.21
MCS Change	0.94	0.02	−3.14	0.0020	0.91	0.98	113	0.08
PCS Change	0.92	0.03	−3.1	0.0020	0.87	0.97	113	0.08
ODI Change	1.10	0.03	3.51	0.0001	1.04	1.16	92	0.13
**Clinical Chart Notes**								
Activities Daily Living	0.68	0.24	−1.09	0.28	0.33	1.37	171	0.01
All Physical Activity	1.34	0.49	0.79	0.43	0.65	2.75	171	0.00
Assistive Devices		AssistiveDevicesBin ! = 0 predicts failure perfectly						
Positive Emphasis	0.14	0.06	−4.81	0.0001	0.062	0.31	171	0.14
Negative Emphasis	1.30	0.81	0.42	0.68	0.38	4.38	171	0.00
Much Emphasis	2.14	0.92	1.77	0.077	0.92	4.99	171	0.02
Semphasis	0.53	0.28	−1.21	0.23	0.19	1.48	171	0.01
All Emphasis	0.19	0.08	−4.18	0.0001	0.088	0.41	171	0.09
All Juxtaposition	2.06	0.76	1.98	0.048	1.01	4.23	171	0.02
All Physical Therapy	1.66	0.67	1.24	0.22	0.74	3.68	171	0.01
All Analgesics	3.14	1.74	2.07	0.038	1.06	9.28	171	0.02
Work	1.00	0.51	0	1.00	0.37	2.70	171	0.00
Pain	1.21	0.52	0.44	0.66	0.52	2.80	171	0.00
Numbness	0.48	0.27	−1.29	0.20	0.16	1.47	171	0.01
Discomfort	4.94	2.68	2.95	0.0030	1.71	14.29	171	0.05
Short Follow-up	1.21	0.44	0.53	0.60	0.59	2.46	171	0.00
Quantified	1.18	0.50	0.38	0.70	0.51	2.69	171	0.00
Prognosis	5.67	2.52	3.91	0.0001	2.38	13.54	171	0.08
Follow-up as necessary	0.10	0.10	−2.22	0.026	0.013	0.76	171	0.05
Incision	1.12	0.40	0.32	0.75	0.56	2.27	171	0.00
ImproveStemmed	2.02	0.75	1.91	0.056	0.98	4.17	171	0.02
Happy	0.20	0.13	−2.57	0.01	0.058	0.68	171	0.05
Encouraged	1.47	0.61	0.92	0.36	0.65	3.31	171	0.00
DenyStemmed	1.20	0.85	0.26	0.79	0.30	4.77	171	0.00
Danger Words		DangerWordsBi n omitted because of collinearity						
Causal	1.06	0.89	0.07	0.95	0.21	5.47	171	0.00
Medium Follow-up	0.91	0.43	−0.2	0.84	0.36	2.30	171	0.00
No further follow-up	5.05	4.71	1.74	0.082	0.81	31.35	171	0.02
Moving goal post	3.28	3.34	1.17	0.24	0.45	24.07	171	0.01
Patient Non Pronoun		PatientNonPronounFinalBin ! = 1 predicts failure perfectly						
Action Plan	1.62	0.64	1.21	0.23	0.74	3.52	171	0.01

## Additional file

Additional file 1:
**Chart Note Node Codes Used in Content Analysis.** (PDF 83 kb)
