# Supplementary material for: When patients and surgeons disagree about surgical outcome: investigating patient factors and chart note communication
Source: Health Qual Life Outcomes. 2015 Sep 29;13:161. doi: 10.1186/s12955-015-0343-0 (PMC4587581; doi:10.1186/s12955-015-0343-0)
Supplement: Additional file 1: — Chart Note Node Codes Used in Content Analysis. (PDF 83 kb) [file 12955_2015_343_MOESM1_ESM.pdf]

Appendix I. Chart Note Node Codes Used in Content Analysis

Variable	Manual or Text Search or Stata	Coded or Empty	Explanation (if necessary)
ActionPlanFinal	Manual	Coded	Prescription or referall to another specialist (usually physiotherapy)
**Activities**	Manual	Empty	
ActivityStemmed	Text Search	Coded	Stemmed search for "activity"
CookingStemmed	Text search	Coded	Stemmed search for "cooking"
Dailyactivitiestogether	Text Search	Coded	"Daily activities"
Daytoday	Text Search	Coded	"Day-to-day"
Everydayactivities	Text Search	Coded	"everyday activities"
**AssistiveDevices**	Manual	Empty	
CanesStemmed	Text Search	Coded	Stemmed search for "canes"
Walker	Text Search	Coded	"Walker"
CausalFinal	Manual	Coded	Something in the clinical note that gives a sense of what is causing the problem
DangerWords	Manual	Empty	
Dare	Text Search	Coded	"Dare"
Executestemmed	Text Search	Coded	Stemmed search for "execute"
DenyStemmed	Text Search	Coded	Stemmed search for "deny"
**DoctorName**	Manual	Empty	
**Finkelstein**	Text Search	Coded	"Finkelstein"
Abbas	Text Search	Coded	"Abbas"
Aleem	Text Search	Coded	"Aleem"
Bronstein	Text Search	Coded	"Bronstein"
Cunha	Text Search	Coded	"Cunha"
Dessouki	Text Search	Coded	"Dessouki"
Johnson	Text Search	Coded	"Johnson"
Khoshbin	Text Search	Coded	"Khoshbin"
Kramer	Text Search	Coded	"Kramer"
lotan	Text Search	Coded	"Lotan"
MedicalStudent	Text Search	Coded	"Medical Student"
Mollon	Text Search	Coded	"Mollon"
Rizek	Text Search	Coded	"Rizek"
Roshkar	Text Search	Coded	"Roshkar"
Shpigelman	Text Search	Coded	"Shpigelman"
Trajkovski	Text Search	Coded	"Trajkovski"
Wasserstein	Text Search	Coded	"Wasserstein"
**Ford**			
Nicholls	Text Search	Coded	"Nicholls"
**Yee**			
Fawaz	Text Search	Coded	"Fawaz"
Haddad	Text Search	Coded	"Haddad"
Kember	Text Search	Coded	"Kember"
Mansouri	Text Search	Coded	"Mansouri"
Miller	Text Search	Coded	"Miller"
DoctorSpeakFinal	Manual	Coded	Usage of medical or technical language

**Emphasis**	Manual	Empty	
Absolutely	Text Search	Coded	"Absolutely"
Atleast	Text Search	Coded	"At least"
Completely	Text Search	Coded	"Completely"
Extremely	Text Search	Coded	"Extremely"
MarkedStemmed	Text Search	Coded	Stemmed search for "marked"
Muchbetter	Text Search	Coded	"Much better"
Muchdifferent	Text Search	Coded	"Much different"
Muchimproved	Text Search	Coded	"Much improved"
Muchmore	Text Search	Coded	"Much more"
Only	Text Search	Coded	"Only"
Significant	Text Search	Coded	"Significant"
Somuch	Text Search	Coded	"So much"
Very	Text Search	Coded	"Very"
Whatsoever	Text Search	Coded	"Whatsoever"
EncouragedFinal	Manual	Coded	Using the word encouraged/suggested/synonym for patient to do certain behaviors
Happy	Text Search	Coded	"Happy"
ImproveStemmed	Text Search	Coded	Stemmed search for "Improve"
Incision	Text Search	Coded	"Incision"
Juxtaposition	Manual	Empty	
Actually	Text Search	Coded	"Actually"
Although	Text Search	Coded	"Although"
But	Text Search	Coded	"But"
However	Text Search	Coded	"However"
Though	Text Search	Coded	"Though"
LongFinal	Manual	Coded	Long follow-up time, seeing a patient based on symptomatology or p.r.n.
**Medications**	Manual	Empty	
**Analgesics**	Manual	Empty	
Arthrotec	Text Search	Coded	"Arthrotec"
Celebrex	Text Search	Coded	"Celebrex"
Gabapentin	Text Search	Coded	"Gabapentin"
Ibuprofen	Text Search	Coded	"Ibuprofen"
Lyrica	Text Search	Coded	"Lyrica"
Motrin	Text Search	Coded	"Motrin"
Oxycodone	Text Search	Coded	"Oxycodone"
Oxycontin	Text Search	Coded	"Oxycontin"
PercocetStemmed	Text Search	Coded	Stemmed search for "percocet"
Tylenol	Text Search	Coded	"Tylenol"
Voltaren	Text Search	Coded	"Voltaren"
**Antibiotics**	Manual	Empty	
AntibioticsStemmed	Text Search	Coded	Stemmed search for "Antibiotics"
Bloodpressuremedicati	Text Search	Coded	"blood pressure medication"

**SubstanceAbuse**	Manual	Empty	
AddictStemmed	Text Search	Coded	Stemmed search for "addict"
MediumFinal	Manual	Coded	Medium follow-up time >6weeks to <1year
MovinggoalpostFinal	Manual	Coded	The patient has a goal, reaches the goal, and then changes the goal
NoneFinal	Manual	Coded	No follow-up; the note specifies the patient does not need to return
PatientNonPronounFin	Manual	Coded	Patient referred to with a word other than a pronoun
**AllPhysicalActivity**	Manual	Empty	
Exercise	Text Search	Coded	"Exercise"
GolfStemmed	Text Search	Coded	Stemmed search for "golf"
PhysicalActivity	Text Search	Coded	"Physical activity"
WalkStemmed	Text Search	Coded	Stemmed Search for "walk"
PhysicalTherapy	Text Search	Coded	"Physical therapy"
Physio	Text Search	Coded	"Physio"
Physiotherapy	Text Search	Coded	"Physiotherapy"
PrognosisFinal	Manual	Coded	Any mention of prognosis (how patients symptoms will change in the future)
QuantifiedFinal	Manual	Coded	Chart note uses numbers to explain something (i.e. patient has 80% less pain…)
ShortFinal	Manual	Coded	Short follow-up time; 6 weeks
**Symptoms**	Manual	Empty	
ConstipationStemmed	Text Search	Coded	Stemmed search for "constipation"
CrampingStemmed	Text search	Coded	Stemmed search for "cramping"
DepressionStemmed	Text Search	Coded	Stemmed search for "depression"
Discomfort	Text Search	Coded	Discomfort
Numbness	Text Search	Coded	Numbness
Pain	Text Search	Coded	Pain
Work	Text Search	Coded	"Work" (in the job sense)
**Bin Suffix**	**BINARY VARIABLE**	**BINARY VARI**	**Bin Suffix**
ActivitiesDailyLiving	Stata	Coded	ActivityStemmedBin + CookingStemmedBin + Dailyactivitiestogetherbin + Daytodaybin + Everydayactivitiesbin
ActivitiesDailyLivingBin	Stata	Coded	
AllPhysicalActivityBin	Stata	Coded	ExerciseBin + GolfStemmedBin + PhysicalActivityBin + WalkStemmedBin
AssistiveDevicesBin	Stata	Coded	CanesStemmedBin + WalkerBin
PositiveEmphasis	Stata	Coded	Absolutely + Whatsoever + Completely + Extremely + Very
PositiveEmphasisBin	Stata	Coded	
NegativeEmphasis	Stata	Coded	Atleast + Only
NegativeEmphasisBin	Stata	Coded	
MuchEmphasis	Stata	Coded	Muchbetter + Muchdifferent + Muchimproved + Muchmore + Somuch
MuchEmphasisBin	Stata	Coded	
Semphasis	Stata	Coded	Significant + MarkedStemmed
SEmphasisBin	Stata	Coded	
AllEmphasis	Stata	Coded	Absolutely + At least + Completely + Extremely + MarkedStemmed + Muchbetter + Muchdifferent + Muchimproved + Muchmore + Somuch + Only + Significant + Very + Whatsoever
AllEmphasisBin	Stata	Coded	
AllJuxtaposition	Stata	Coded	Actually + Although + But + However + Though
AllJuxtapositionBin	Stata	Coded	
AllPhysicalTherapy	Stata	Coded	PhysicalTherapy + Physio + Physiotherapy
AllPhysicalTherapyBin	Stata	Coded	
AllAnalgesics	Stata	Coded	Arthrotec + Celebrex + Gabapentin + Ibuprofen + Lyrica + Motrin + Oxycodone + Oxycontin + PercocetStemmed + Tylenol + Voltaren
AllAnalgesicsBin	Stata	Coded	
